# Real‐world use of continuous glucose monitoring in people with type 2 diabetes and chronic kidney disease or on dialysis

**DOI:** 10.1111/dme.70174

**Published:** 2025-11-18

**Authors:** Patrick McGown, Nikoleta Papanikolaou, Jasmine El‐Taraboulsi, Clemency Britton, Javeria Iqtidar, Khuram Chaudhry, Shiyan Ng, Kishan Jethwa, Jagvi Patel, Jo Reed, Monika Reddy, Lalantha Leelarathna, Andrew H. Frankel, Chukwuma Uduku, Parizad Avari

**Affiliations:** ^1^ Department of Diabetes and Endocrinology Imperial College London NHS Trust London UK; ^2^ Department of Renal Medicine Imperial College London NHS Trust London UK; ^3^ Department of Metabolism, Digestion and Reproduction Imperial College London London UK

**Keywords:** chronic kidney disease, continuous glucose monitoring, hypoglycaemia, type 2 diabetes

## Abstract

**Introduction:**

Continuous glucose monitoring (CGM) has become the standard of care for people with type 1 diabetes (T1D) and some people with type 2 diabetes (T2D). However, there is a lack of data regarding CGM use in people with T2D and chronic kidney disease (CKD), who are at increased risk of hypoglycaemia. We assess the use of CGM and glucose outcomes in this cohort.

**Methods:**

Retrospective, observational analysis of adults on CGM attending a tertiary diabetes renal clinic between January 2023 and September 2024. People with T2D on multiple daily insulin injections (defined as 2 or more insulin injections per day) and CKD stage 3–5 (eGFR <60, on renal replacement therapy or post‐renal transplant) were included. HbA1c was assessed pre‐ and post‐initiation of CGM. CGM metrics were analysed for percentage times in ranges and other glycaemic metrics.

**Results:**

A total of 177 adults (median (interquartile range)) aged 64.0 (56.0–71.0) years were included. Time below range (<3.9 mmol/L) was significantly reduced (median (interquartile range)) from 1.0 (0.0–2.0)% to 0.0 (0.0–1.0)% following CGM initiation (*p* = 0.007). Hypoglycaemia leading to an insulin dose reduction was revealed by CGM in 91/177 participants (51.4%). Median HbA1c pre‐ and post‐CGM was 64.0 (53.0–79.0) mmol/mol (8.0, 7.0–9.4%) and 62.0 (51.8–73.0) mmol/mol (7.8, 6.9–8.8%), respectively (*p* < 0.001). HbA1c reduction was observed in 114/177 participants (64.4%).

**Conclusion:**

CGM use was associated with the identification of hypoglycaemia and improvements in HbA1c in people with T2D and CKD. These real‐world data demonstrate the importance of CGM technology to high‐risk individuals with diabetes and CKD.

**What's new?**

- Continuous glucose monitoring (CGM) has become the standard of care for people with type 1 diabetes; however, its use in people with moderate/advanced CKD and dialysis remains a key research area.
- This study demonstrates that the use of CGM reduces the incidence of hypoglycaemia in people with type 2 diabetes and CKD.
- These real‐world data are important for supporting the access to CGM for people at high risk of hypoglycaemia from CKD.

## 1 INTRODUCTION

Continuous glucose monitoring (CGM) has become the standard of care for people with type 1 diabetes (T1D)^1^ and some people with type 2 diabetes (T2D).^2^ The International Consensus Guidance on CGM recommends that high‐risk populations with diabetes aim for >50% time in range (TIR; 3.9–10 mmol/L) with <1% time below range (TBR; <3.9 mmol/L).^3^

Data from people with T1D demonstrate that CGM in the outpatient setting improves glycaemia,^4^, ^5^ increases quality of life^6^ and reduces admissions with diabetic emergencies.^7^ In the United Kingdom, the National Institute of Clinical Excellence (NICE) recommends the use of CGM for people with T2D on multiple daily insulin injections if they have recurrent hypoglycaemia or severe hypoglycaemia, impaired hypoglycaemia awareness, a condition or disability, which means they cannot self monitor their blood glucose by capillary glucose monitoring, would otherwise be advised to self‐measure at least eight times a day, or if they would need help from a care worker or healthcare professional to monitor their blood glucose.^2^ At the time of writing, despite the above guidance from NICE, many local integrated care boards have not yet adopted or implemented the guidance for people with T2D.

T2D is the most common cause of end‐stage kidney disease (ESKD) in developed countries,^8^ with glucose management in ESKD being particularly complex due to disruptions in glucose homeostasis. People with diabetes and advanced kidney disease have the highest rates of hospital admissions with hypo‐ and hyperglycaemic emergencies, wide glucose fluctuations, high mortality and low quality of life.^9^, ^10^ Chronic kidney disease (CKD) is also an independent risk factor for hypoglycaemia.^11^ Hypoglycaemia is a cause of significant morbidity and mortality in diabetes,^12^ and it is also acknowledged that fear of hypoglycaemia is a barrier to optimal glycaemic control.^13^

In CKD, biomarkers including HbA1c, the gold‐standard indicator of glycaemic control, can be unreliable due to a wide variety of factors. Confounders include shortened red cell lifespan, anaemia, the haemodialysis process, treatment with erythropoietin stimulation, red blood cell transfusions and uraemia.^14^ These lead to an overall underestimation of plasma glucose by HbA1c in advanced CKD, when compared to CGM or alternative biomarkers such as fructosamine or glycated albumin.^14^ Alternative biomarkers are not recommended for use by major guidelines, and indeed have their own drawbacks such as lack of availability in real‐world settings, lack of established treatment goals, reduced glycaemic assessment periods and bias in the presence of hypoalbuminaemia, proteinuria or peritoneal dialysis.^15^

An observational study in people with diabetes and CKD on haemodialysis demonstrates that CGM may be a more appropriate measure of glycaemia than HbA1c^16^ and may represent a more convenient method of monitoring than traditional methods.^15^ Various accuracy studies have been conducted demonstrating suitable accuracy of CGM in advanced CKD and ESKD.^17^, ^18^ However, evidence detailing the benefits of CGM in this cohort is lacking,^19^ and the use of technology in people with moderate/advanced CKD and dialysis remains a key research area.^20^

The aims of this real‐world analysis were to evaluate the safety, efficacy and utility of CGM in people with T2D and CKD, in relation to glycaemic outcomes and identification of hypoglycaemia.

## 2 METHODS

### 2.1 Study population and data collection

This was a retrospective, observational study. People aged >18 years on CGM attending a tertiary diabetes renal clinic between January 2023 and September 2024 were identified with the use of electronic medical records.

Data were obtained for adults with T2D on multiple daily insulin injections (defined as 2 or more insulin injections per day) and renal impairment, defined using KDIGO criteria as CKD stage ≤3 (eGFR <60, on renal replacement therapy or renal transplant).^21^ Those with T1D or type 3c diabetes were excluded as they are already eligible for CGM in the United Kingdom as per the NICE guidelines.^1^

The study was entirely observational (with no deviation from standard clinical care), and ethics approval was not required. All individuals had granted specific permission to share their glucose data with the clinical teams and for clinic staff to access this, when linking their devices and uploading their data to web‐based online software (LibreView and Dexcom Clarity). NHS Research Ethics Committee review was not required. Demographic (age, sex, self reported ethnicity), clinical (date of diagnosis, technology use) and biochemical data (HbA1c on initiation and 6 months post initiation) were collected for all eligible individuals.

CGM metrics were collected at initiation for 28 days and on follow‐up. CGM data were obtained for glucose management indicator (GMI), glucose variability (defined as percent coefficient of variation (%CV)), active sensor time and the percentage of time spent within the CGM device's categorical glucose ranges (‘Very high’ [>13.9 mmol/mol]; ‘High’ [10.1–13.9 mmol/mol]; ‘Time in range’ [TIR, 3.9–10.0 mmol/mol]; ‘Low’ [3.0–3.8 mmol/mol]; ‘Very low’ [<3.0 mmol/mol]). Clinic notes were also reviewed to determine whether any insulin dose reduction was actioned as a response to identified hypoglycaemia, and to find the documented reason for CGM initiation.

Socio‐economic deprivation was assessed by the English Indices of Deprivation 2019. Deprivation deciles are based on the Index of Multiple Deprivation (IMD) 2019.^22^ Decile 1 represents the most deprived 10% of neighbourhoods in England, while decile 10 represents the least deprived 10%.

### 2.2 Statistical analysis

Baseline characteristics are presented as median (interquartile range) for continuous data and as frequencies of counts (percentages) for categorical data. Wilcoxon rank testing was performed to assess for differences between paired readings (initial and follow‐up) for HbA1c and CGM metric data. Results are presented as median (interquartile range) due to the asymmetric nature of CGM glycaemic data. An overall *p*‐value of <0.05 was considered statistically significant, with the Holm correction for multiple analyses used to adjust the *p*‐value significance threshold depending on the number of tests performed. Statistical analysis was performed using the Prism statistical package (GraphPad Software Inc., San Diego, USA).

## 3 RESULTS

### 3.1 Demographics

A total of 570 people with diabetes and CKD were reviewed in the diabetes renal clinic within the time frame studied. People with T1D or type 3c diabetes were excluded, and the remaining were screened for eligibility as per the KDIGO renal impairment criteria.

Subsequently, a total of 202 adults with T2D and CKD were offered CGM within this period. Two individuals who provided permission for CGM during their clinic appointment were deceased prior to initiation (where CGM start had been scheduled for a later date). Four participants were excluded from the analysis due to the absence of follow‐up HbA1c or CGM data beyond three months. Additionally, 19 people were offered CGM but declined. Thus, 177 adults were included in the final analysis.

Baseline demographics (Table 1) demonstrate 64% were male, with a median (IQR) age of 64 (56–71) years. Median eGFR was 32 (18–46) ml/min/1.73m^2^, with 28 individuals (16%) on dialysis.

**TABLE 1:** Population demographics.

Participants	*n* = 177
Age, years	64 (56–71)
Sex	
Male	114 (64%)
Female	63 (36%)
Diagnosis	
T2D	143 (81%)
PTDM	34 (19%)
Median eGFR (ml/min/1.73m^2^)	32 (18–46)
eGFR <30	85 (48%)
ESKD on dialysis	28 (16%)
Ethnicity	
Black	25 (14%)
Indian, Pakistani, Bangladeshi	54 (31%)
Other Asian	18 (10%)
White	35 (20%)
Any other ethnicity	19 (11%)
Not stated	23 (13%)
Index of Multiple Deprivation Decile (Most deprived: 1, Least deprived: 10)	4 (3–6)

*Note*: Table 1 showing population demographics. Data are presented as *n* (%) or median (IQR).

Abbreviations: eGFR, estimated glomerular filtration rate; ESKD, end‐stage kidney disease; PTDM, post‐transplant diabetes mellitus; T2D, type 2 diabetes mellitus.

### 3.2 Glycaemic outcomes

**FIGURE 1:**
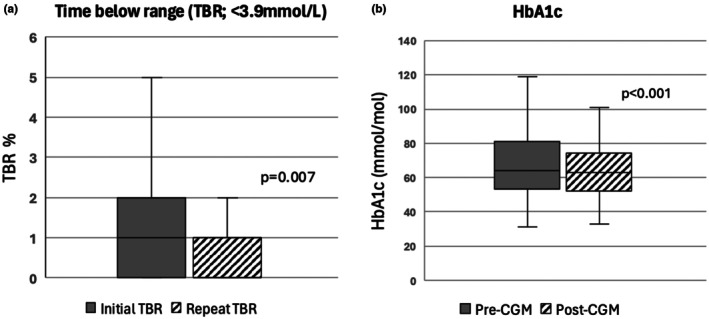
Box‐plots demonstrating (a) time below range (<3.9 mmol/L) on initiation and post‐6 months of CGM use (b) HbA1c pre‐ and post‐CGM initiation. Box‐plots demonstrating (a) time below range (<3.9 mmol/L) on initiation and post‐6 months of CGM use (b) HbA1c pre‐ and post‐CGM initiation. TBR, time below range. Outer whiskers of the box plot extend to the most extreme values within 1.5 times the interquartile range. A *p* value <0.05 indicates statistical significance.

Following CGM initiation, there was a significant reduction (median (IQR)) in time below range (TBR, <3.9 mmol/L) from 1.0 (0.0–2.0)% to 0.0 (0.0–1.0)% (*p* = 0.007; Table 2; Figure 1a). The reasons for CGM initiation documented in the clinical notes included: high risk of hypoglycaemia due to renal impairment on multiple injections (*n* = 92), recurrent hypoglycaemia (*n* = 18), unable to self monitor (*n* = 9), advised to monitor at least eight times per day (*n* = 6) and lack of hypoglycaemic awareness (*n* = 4). In 91 of 177 individuals (51.4%), CGM identified episodes of hypoglycaemia that led to a reduction in total daily dose of insulin (including both simple dose reductions and regimen changes).

**TABLE 2:** Glycaemic data on initiation and post‐6 months of CGM use.

	CGM on initiation	CGM after 6 months of use	*p*‐value
Time below range (%) (<3.9 mmol/L)	1.0 (0.0–2.0)	0.0 (0.0–1.0)	0.007*
Low (%) (3.0–3.8 mmol/L)	2.0 (0.0–2.0)	1.0 (0.0–1.0)	0.006*
Very low (%) (<3.0 mmol/L)	0.0 (0.0–0.0)	0.0 (0.0–0.0)	0.119
Time in range (%) (3.9–10.0 mmol/L)	61.0 (37.5–78.0)	56.0 (39.0–76.0)	0.343
Time above range (%) (>10.0 mmol/L)	36.5 (19.0–60.3)	43.0 (22.8–61.0)	0.197
High (%) (10.1–13.3 mmol/L)	25.0 (16.0–32.5)	28.0 (19.0–35.0)	0.004*
Very high (%) (>13.3 mmol/L)	8.0 (1.5–25.5)	10.0 (1.0–23.0)	0.995
CV (%)	32.7 (28.0–36.6)	30.4 (26.9–35.2)	<0.001*
HbA1c (mmol/mol)	64.0 (53.0–79.0)	62.0 (51.8–73.0)	<0.001*
HbA1c (%)	8.0 (7.0–9.4)	7.8 (6.9–8.8)	
CGM‐estimated A1c	57.0 (51.0–65.0)	58.0 (52.0–63.8)	0.241

*Note*: Results presented as median (IQR). Table showing Glycaemic data on initiation and post‐6 months of CGM use. Results presented as median (IQR). *denotes significance at *p* = 0.01. Using the Holm statistical method to correct for multiple analyses, the adjusted threshold of statistical significance is first rejected at *p* = 0.01.

There was no significant change in TIR following CGM initiation. Percentage time in the high range increased from 25.0 (16.0–32.5)% to 28.0 (19.0–35.0)% (*p* = 0.004); however, there was no significant increase in time in the very high range or overall TAR. Median sensor wear time was 70.5 (43.0–89.0)% on initiation and 88.5 (61.0–98.0)% on follow‐up (*p* < 0.001).

CV reduced from 32.7 (28.0–36.6)% to 30.4 (26.9–35.2)% (*p* = 0.002).

Median HbA1c prior to CGM use was 64.0 (53.0–79.0) mmol/mol (8.0, 7.0–9.4%) and median HbA1c on repeat testing following CGM for 6–12 months was 62.0 (51.8–73.0) mmol/mol (7.8, 6.9–8.8%) (*p* < 0.001) (Figure 1b; Table 2).

Sub‐analysis of people with advanced CKD (eGFR <30) and those on dialysis showed similar findings. For people on dialysis, TBR <3.9 mmol/L was 1.0 (0.0–3.0)% at initiation and TBR 0.0 (0.0–1.06)% after 6 months (*p* = 0.085), demonstrating a trend for reducing hypoglycaemia, although numbers were too small to reach statistical significance.

### 3.3 Discontinuation/Safety

Nine people (5.1%) discontinued CGM during the study period. Reasons for discontinuation included dislike of scanning (*n* = 3), alarm fatigue (*n* = 2), device failure (*n* = 2), falsely low readings (*n* = 1) and cognitive issues (*n* = 1). Data from those who discontinued were excluded from analysis if no CGM data were available or if there was no repeat HbA1c (*n* = 4).

## 4 DISCUSSION

This retrospective, cohort study demonstrates the utility and safety of CGM in people with CKD, including those receiving dialysis. We observed a statistically significant reduction in TBR, reflecting a meaningful decrease in hypoglycaemic burden, with subsequent adjustments in insulin dosage.

The use of CGM offers advantages in this high‐risk population. People with CKD, particularly those with ESKD on dialysis, face unique challenges in glucose management due to altered insulin metabolism, reduced renal gluconeogenesis and the impact of dialysis on glucose variability. These factors contribute to a heightened risk of hypoglycaemia, which is associated with increased risks of both mortality and hospitalisation.^9^, ^23^, ^24^

A significant improvement in HbA1c was also observed with the use of CGM. This may be attributed to more precise insulin titration guided by patterns identified through CGM data, as well as a reduction in overcorrection of glucose levels following fewer episodes of hypoglycaemia. These factors likely contributed to a reduction in glycaemic variability, as observed in our cohort. Despite this, there was no significant difference in the CGM‐estimated A1c in this study. Possible explanations include variable active sensor time, inaccuracy of HbA1c in this cohort, and that different time periods were assessed between the two metrics. Indeed, the CGM estimated A1c values will likely underestimate the utility of CGM as the baseline data includes 28 days of CGM use.

The overall benefits of reduced hypoglycaemia are likely from real‐time data provided by CGM, which enables the user to take action following alarms and trend arrows for impending (predictive) and established hypo‐ and hyperglycaemia. The ability of CGM to detect asymptomatic and nocturnal hypoglycaemia is particularly valuable in this context, where standard monitoring may fail to capture clinically significant glycaemic excursions.

Accuracy of CGM systems for people with ESKD and on dialysis has previously been a concern due to several physiological factors affecting this population, including fluid shifts during dialysis, uraemia and changes in tissue perfusion, impacting sensor function and glucose diffusion in the interstitial space. Recent studies have highlighted sufficient CGM accuracy in this cohort.^17^, ^18^, ^25^

The strengths of this study include an ethnically diverse study population, and the generation of real‐world CGM data in an understudied population with T2D and CKD. Whilst use of CGM increases costs per person year, this is typically offset by the larger cost for people with type 2 diabetes who have an episode of severe hypoglycaemia than those with type 1 diabetes.^26^ Savings in the reduction of daily strips and lancets somewhat offset the sensor system acquisition cost, and a Spanish study looking at the use of the Freestyle Libre 2 system in people with type 2 diabetes found that it resulted in a net saving versus self‐monitoring of blood glucose.^27^ Additional savings would be made via reduction of cardiovascular events associated with hypoglycaemia (HR 1.9), including mild hypoglycaemia (HR 1.68), based on meta‐analysis data.^28^ Furthermore, the incidence of hypoglycaemia is greater in people on dialysis^29^ and this is likely to amplify the benefit.

However, there are several limitations; first, the absence of a control group using traditional glucose monitoring limits our ability to make direct comparisons or infer causality. Whilst changes in TBR and HbA1c are encouraging, we cannot exclude the influence of other factors such as clinical contact, concurrent medication adjustments or behavioural changes. (Note: Tirzepatide, a novel dual GIP/GLP‐1 receptor agonist, was not available for use within our Trust until late July 2024; therefore, no individuals in the study population would have received this therapy. Additionally, there was a national supply shortage of GLP‐1 receptor agonists during the study period,^30^ making it unlikely that any observed treatment effects were attributable to this class of medication). We did not formally assess user satisfaction or quality of life, which are important considerations when evaluating the overall impact of CGM in this population, and we did not have a robust method for collecting data on hypoglycaemic admissions or ambulance call outs during the study period (our electronic health care records were not linked to other district general hospitals in our area at the time). Furthermore, a minority of participants declined CGM or discontinued use due to factors such as alarm fatigue, device‐related concerns or user burden. These barriers highlight the importance of user education, appropriate device selection and ongoing support to promote sustained engagement. Finally, studies have demonstrated relatively rapid improvements with CGM, and our baseline data are already affected by CGM use for 28 days; therefore, this study likely underestimates the true benefits of CGM.

## 5 CONCLUSION

The use of CGM was associated with a significant reduction in hypoglycaemia among adults with T2D and CKD. These real‐world data are important for supporting broader access to CGM for people with CKD, a group at heightened risk of hypoglycaemia and associated complications. Further studies with formal health economic analyses should be conducted.

## CONFLICT OF INTEREST STATEMENT

P.A. has received equipment from Dexcom for investigator‐initiated studies. L.L. has received speaker honoraria from Abbott Diabetes Care, Insulet, Medtronic, Novo Nordisk, Roche and Sanofi; was on advisory panels for Abbott, Novo Nordisk, Dexcom, Medtronic, Sanofi and Roche; and received research support from Novo Nordisk, Abbott Diabetes Care and Dexcom. M.R. has received honoraria for advisory board participation from Dexcom and Roche Diabetes. A.F. has received honoraria for speaking and advisory board participation from Astra Zeneca, Boehringer Ingelheim, Lilly, Napp UK and Vifor Pharma UK.
